# Acquired nonobstructive urinary bladder diverticulum: a case report

**DOI:** 10.1186/1757-1626-2-36

**Published:** 2009-01-09

**Authors:** Plamen Yovchevski, Kosta Kostov

**Affiliations:** 1 Nephrological Department, Medical Institute Ministry of Interior, Sofia, Bulgaria; 2 Neurological Department, Medical Institute Ministry of Interior, Sofia, Bulgaria

## Abstract

**Introduction:**

Urinary bladder diverticula are frequently resulting from obstructions. Our literature review did not reveal any cases of acquired urinary bladder diverticulum caused by long-term transurethral catheterization.

**Case Presentation:**

We report a rare case of a nonobstructive big urinary bladder diverticulum developed after a long-term urethral catheterization in a 62-year old male diabetic patient with normal subvesical urinary tract. The diverticulum was demonstrated by ultrasonography. Its formation was associated with the decubital changes of the bladder wall when the Foley catheter stayed for a longer period.

**Conclusion:**

Ultrasonographic examination of the urinary bladder is necessary to exclude such complication after a long-lasting catheterization as well as to maximally restrict the catheter's stay in the urinary bladder.

## Introduction

Bladder diverticula are herniations of the bladder mucosa and submucosa through bladder muscular wall. They often cause no symptoms and are incidentally discovered during examination for other reasons. But sometimes patients present with urinary tract infections caused by diverticula, especially when they are large and empty poorly. Stasis of urine within diverticula can also lead to stone formation or epithelial dysplasia [1]. Acquired diverticula are frequently resulting from obstructions, and rarely from iatrogenic causes like surgical interventions. However, our literature review did not reveal any cases of acquired urinary bladder diverticulum caused by long-term catheterization. Transurethral catheterization of the urinary bladder is usually accompanied by other fast developing complications – urinary tract infections [2], epididymo-orchitis [3], hemorrhage [4] and perforation [5].

The purpose of our study is to report the case of a patient with acquired nonobstructive urinary bladder diverticulum which was diagnosed with power Doppler sonography on routine ultrasonographic examination. We also aim to highlight the potential role of ultrasonographic examination as a noninvasive diagnostic method for such problems of the lower urinary tract.

## Case Presentation

**Figure 1:**
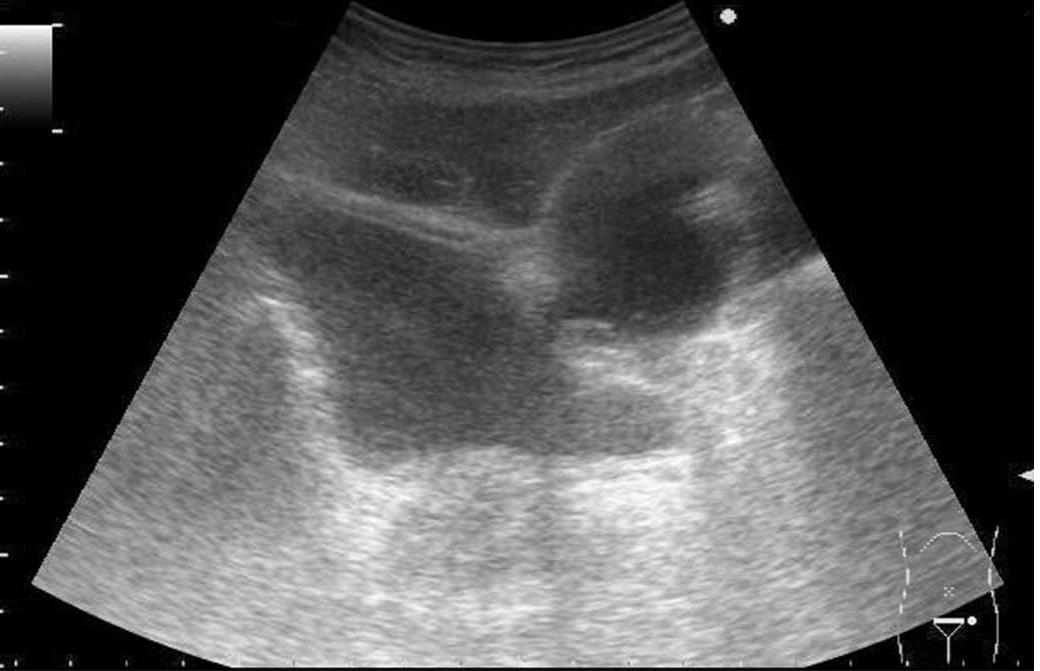
**Transverse transabdominal grayscale sonogram of the pelvis showing a large bladder diverticulum**.

**Figure 2:**
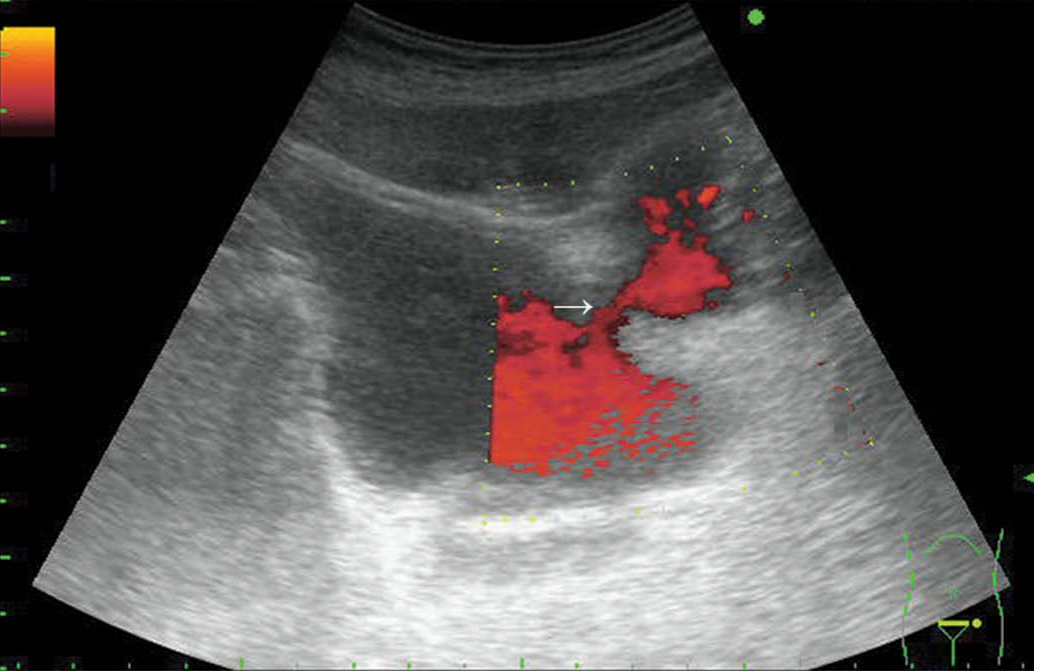
**Power Doppler sonography revealing a communication (arrow) between urinary bladder and diverticulum**.

A 62-year old male patient with diabetes mellitus (DM) type 1 underwent series of surgical interventions after a car accident which necessitated long-term urethral catheterization. The Ultrasonographic and Computer Tomographic examinations of the patient's abdomen after the accident showed intact urinary bladder and no obstructions in the subvesical urinary tract. A 3-month urethral catheterization was applied because of the long surgical and orthopedic treatment. The catheter was replaced by a new one every 30 days in this 3-month period. The past medical history of the patient included only diabetes mellitus type 1 since the age of 9. 1 month after the complete removal of the catheter a routine urinary tract sonography was performed. Transabdominal grayscale and power Doppler sonography was performed using a Prosound SSD-3500 scanner (Aloka Ltd., Tokyo, Japan) equipped with a convex array transducer 3.5 MHz (UST 979). Transabdominal grayscale (Figure 1) showed a large diverticulum protruding from the urinary bladder. Power Doppler sonography (Figure 2) revealed a communication (arrow) between the urinary bladder and the diverticulum.

## Discussion

The long-term stay of the catheter may cause a damage of the uroepithelial layer not only of the urethra but also of the opposite bladder wall (the one located opposite to the tip of the catheter). This problem may relatively more often occur in diabetic patients without any symptoms and may reveal itself in a later period. In our patient the chronological examination of the clinical data showed a causal relationship between the catheterization and the appearance of the diverticulum. This is a rare late complication of the urethral catheterization, which necessitates an ultrasonographic examination of the lower urinary tract after the complete catheter removal. A thorough examination of the clinical indications for Foley catheter placement and its timely removal should be conducted. This is very important for patients with DM, who are prone to more frequent and serious complications resulting from the impaired sensitivity, which is caused by diabetic polyneuropathy. This leads to a pain absence at urinary bladder wall damages. We believe this to be the first recorded case of acquired nonobstructive urinary bladder diverticulum diagnosed via power Doppler sonography. Our case demonstrates that the development of ultrasonography has led to the possibility of a more precise evaluation of lower urinary tract. Bladder ultrasonography is a good first-line imaging method for discovering bladder diverticula because anteriorly and posteriorly placed diverticula may not be noticed when voiding cystourethrography is used as they are overshadowed by the contrast in the bladder.

## Abbreviations

MHz: Megahertz; DM: Diabetes mellitus.

## Consent

Written informed consent was obtained from the patient for publication of this case report and accompanying images. A copy of the written consent is available for review by the Editor-in-Chief of this journal.

## Competing interests

The authors declare that they have no competing interests.

## Authors' contributions

Both authors participated in the care of this patient and contributed to the manuscript. Both authors have red and approved the final version of the paper.
